# Mutagenesis and Functional Studies with Succinate Dehydrogenase Inhibitors in the Wheat Pathogen *Mycosphaerella graminicola*

**DOI:** 10.1371/journal.pone.0035429

**Published:** 2012-04-19

**Authors:** Gabriel Scalliet, Judith Bowler, Torsten Luksch, Lucy Kirchhofer-Allan, Diana Steinhauer, Keith Ward, Michael Niklaus, Andreas Verras, Michael Csukai, Antoine Daina, Raymonde Fonné-Pfister

**Affiliations:** 1 Syngenta Crop Protection Münchwilen AG, Stein, Switzerland; 2 Syngenta Jealott's Hill International Research Center, Bracknell, United Kingdom; Université Paris-Sud, France

## Abstract

A range of novel carboxamide fungicides, inhibitors of the succinate dehydrogenase enzyme (SDH, EC 1.3.5.1) is currently being introduced to the crop protection market. The aim of this study was to explore the impact of structurally distinct carboxamides on target site resistance development and to assess possible impact on fitness.

We used a UV mutagenesis approach in *Mycosphaerella graminicola*, a key pathogen of wheat to compare the nature, frequencies and impact of target mutations towards five subclasses of carboxamides. From this screen we identified 27 amino acid substitutions occurring at 18 different positions on the 3 subunits constituting the ubiquinone binding (Qp) site of the enzyme. The nature of substitutions and cross resistance profiles indicated significant differences in the binding interaction to the enzyme across the different inhibitors. Pharmacophore elucidation followed by docking studies in a tridimensional SDH model allowed us to propose rational hypotheses explaining some of the differential behaviors for the first time. Interestingly all the characterized substitutions had a negative impact on enzyme efficiency, however very low levels of enzyme activity appeared to be sufficient for cell survival. In order to explore the impact of mutations on pathogen fitness *in vivo* and *in planta*, homologous recombinants were generated for a selection of mutation types. *In vivo*, in contrast to previous studies performed in yeast and other organisms, SDH mutations did not result in a major increase of reactive oxygen species levels and did not display any significant fitness penalty. However, a number of Qp site mutations affecting enzyme efficiency were shown to have a biological impact *in planta*.

Using the combined approaches described here, we have significantly improved our understanding of possible resistance mechanisms to carboxamides and performed preliminary fitness penalty assessment in an economically important plant pathogen years ahead of possible resistance development in the field.

## Introduction

*Mycosphaerella graminicola* (Fückel) J. Schröt. in Cohn (anamorph *Septoria tritici*) is the causal agent of Septoria leaf blotch (STB), a major threat to bread and durum wheat worldwide [1], [2], [3]. Breeding programs are ongoing to stack host resistance genes and create new varieties highly resistant to STB, but current control of this fungal disease relies heavily on fungicide use. Options for the chemical control of STB were decreased recently after the rapid development of resistance against the QoI fungicides in this pathogen [4], [5], [6]. For QoIs, the resistance mechanism was attributed to target site mutations in the *Cytb* gene of the target enzyme Cytochrome C reductase also known as the respiratory channel complex III [7]. Mainly, two amino acid substitutions namely F129L and G143A in the mitochondrial CytB protein were detected in field pathogens and these substitutions are responsible for the dramatic loss of efficacy observed for this whole class of fungicides. Currently, the great majority of the European *M. graminicola* population carries the G143A mutation, making its control highly reliant on C14-demethylase inhibitors (DMI) usage targeting the ergosterol biosynthesis pathway and on the multisite fungicide chlorothalonil (CTN). Gradual shifts in DMI sensitivity observed as an incremental reduction in sensitivity of pathogen population towards DMIs over time [8] further stress the importance of introducing novel modes of action for STB control. The availability of compounds with different modes of action is an essential component for effective anti-resistance strategies contributing to wheat yield security [9].

The introduction of novel carboxamide fungicides has delivered a major mode of action to almost all fungicide market segments including fruits, vegetables and cereals. These molecules display fungicidal activity by disrupting the mitochondrial tricarboxylic acid cycle (TCA) through inhibition of the succinate dehydrogenase (SDH) enzyme (also called succinate ubiquinone oxidoreductase (SQR), EC 1.3.5.1). The official term, as stated by the Fungicide Resistance Action Committee (www.FRAC.info) for this fungicidal class is “SDHIs” for succinate dehydrogenase inhibitors. At the molecular level, carboxamides inhibit ubiquinone reduction by binding to the ubiquinone binding site (Qp site) of the SDH enzyme [10]. The SDH enzyme is composed of four polypeptides which are nuclear encoded. SDHA and SDHB subunits assemble into the so called soluble catalytic dimer which faces the matrix whereas SDHC and SDHD subunits form the integral membrane component anchoring the heterotetrameric enzyme to the internal membrane of the mitochondria. Catalytic mechanisms by which electrons are transferred from succinate to ubiquinone involve: (i) oxidation of succinate at the level of SDHA which carries a covalent FAD (ii) transfer of electrons through the iron sulfur clusters [2Fe-2S], [4Fe-4S], and [3Fe-4S] carried by the SDHB subunit, (iii) two step reduction of the ubiquinone at the so called Qp site formed by the interface of SDHB SDHC and SDHD subunits. This later reaction involves transient formation of a semi quinone radical and the intervention of a heme which forms an integral part of the complex [11], [12], [13]. Crystal structures of the enzyme have been resolved for *Escherichia coli* [14], *Gallus gallus* (chicken) [15] and *Sus scrofa* (pig) [16].

Carboxin, was the first carboxamide to be developed for crop protection and was used as seed treatment displaying mainly a basidiomycete spectrum of control [17], [18]. Continuous research has led to the discovery of new chemical structures which modified and broadened this initial narrow biological spectrum and improved potency to the levels required from a modern fungal control agent. Newly discovered molecules include Penthiopyrad (Mitsui chemicals), Boscalid (BASF), Bixafen (Bayer), Fluopyram (Bayer), Sedaxane (Syngenta) and Isopyrazam (Syngenta), some of which display outstanding performance for STB control in the field. Even though SDHIs will be used in mixtures with a maximum of two applications per season in order to minimize the resistance development risk (FRAC), the almost simultaneous introduction of compounds displaying similar modes of action will impose a significant selection pressure on *M. graminicola* populations, in particular within European regions of high wheat production [19]. This further stresses the importance of understanding possible resistance mechanisms to better predict the emergence, spread and persistence of resistance to this class of fungicides in order to develop effective resistance monitoring and anti resistance strategies [20]. A number of target mutations have already been described both in the lab and in the field which can lead to carboxamide resistance. Artificial mutants with amino acid substitutions in the *SDH* genes encoding the Qp site of SDH have previously been produced in various fungal species including *Ustilago maydis* (B_H257L) [21], [22], *M. graminicola* (SDHB_H267L/Y) [23], and more recently at various loci in *Aspergillus oryzae* (SDHB_H249Y/L/N, SDHC_T90I, SDHD_D124E) [24]. A spontaneous mutant conferring Flutolanil and Carboxin resistance was also reported and characterized in *Coprinus cinereus* (SDHC_N80K) [25]. In all these studies functional confirmation was obtained by expression of the mutated alleles in the WT background. In fact it has been suggested that these mutant genes may provide dominant selection markers that can be used in many commercially relevant fungal species [24], [26], [27]. Resistance towards Carboxin was claimed for barley field isolates of *Ustilago nuda* in France, Canada and Italy and the resistance mechanism although not elucidated was reported as monogenic [28], [29]. More recently, target site mutations which confer Boscalid resistance have been detected in various species in the field including *Botrytis cinerea* [30], [31], [32] and *Alternaria alternata* [33], [34]. Complex cross resistance patterns, including negative cross resistance were reported for Oxathiin Carboxamides in the late nineteen seventies [35]. Recent investigations performed with highly Boscalid resistant field isolates of *Corynespora cassiicola* and *Podosphaera xanthii* show that striking lack of cross resistance can be found across novel carboxamides [36]. A recent cross resistance study performed with a range of novel *M. graminicola* mutants which were selected on Carboxin only confirmed this is also true in *M. graminicola* [37]. This suggests that commercially introduced carboxamide SDHIs differ in their binding properties to the SDH enzyme.

The primary aim of this study was to understand possible target site resistance mechanisms to a range of newly introduced subclasses of carboxamides in *M. graminicola* by exploring the impact of target mutations on compound binding, enzyme efficiency and pathogen fitness. To this end *M. graminicola* was subjected to random UV mutagenesis and 5 structurally distinct carboxamides were used for selection. The characterization of more than 480 mutants enabled the identification of as many as 27 substitution types affecting in total 18 positions in the SDHB, SDHC and SDHD-Qp site forming proteins. Characterization of the mutants enabled the identification of substitution types that display selectivity to structurally distinct carboxamides. Docking studies, using a homology model of *M. graminicola* SDH, offer valuable insight to some of the experimental findings. Characterization of enzyme efficiencies showed that the ubiquinone reduction step was impaired in all mutants. Using transgenic strains expressing mutated copies of the enzyme SDH subunits we showed that very low levels of SDH activity is required for the establishment of resistance *in vivo*. Finally, homologous recombinant gene replacements for the most relevant substitutions types enabled preliminary fitness studies *in vitro* and *in planta* to be performed. Homologous recombinant strains developed in this haploid pathogen, correspond to the introduction of a single mutation in the whole genome enabling us to perform a very clean comparison of likely biochemical factors affecting fitness. Using these homologous recombinant strains we unexpectedly found that *M. graminicola* Qp site mutations did not significantly impact reactive oxygen species (ROS) production *in vivo*. However, *in planta* virulence was affected suggesting that these carboxamide selected Qp site mutations have an impact on the biology of this pathogen. Our study nicely complements recent results reported with *M. graminicola* Carboxin-selected mutants [37], whilst our modelling approach enables us to propose a more accurate model of the binding interaction which fits all our more extensive experimental findings for this class of inhibitors.

## Results

### Generation of *M. graminicola* mutants resistant to carboxamides

**Figure 1:**
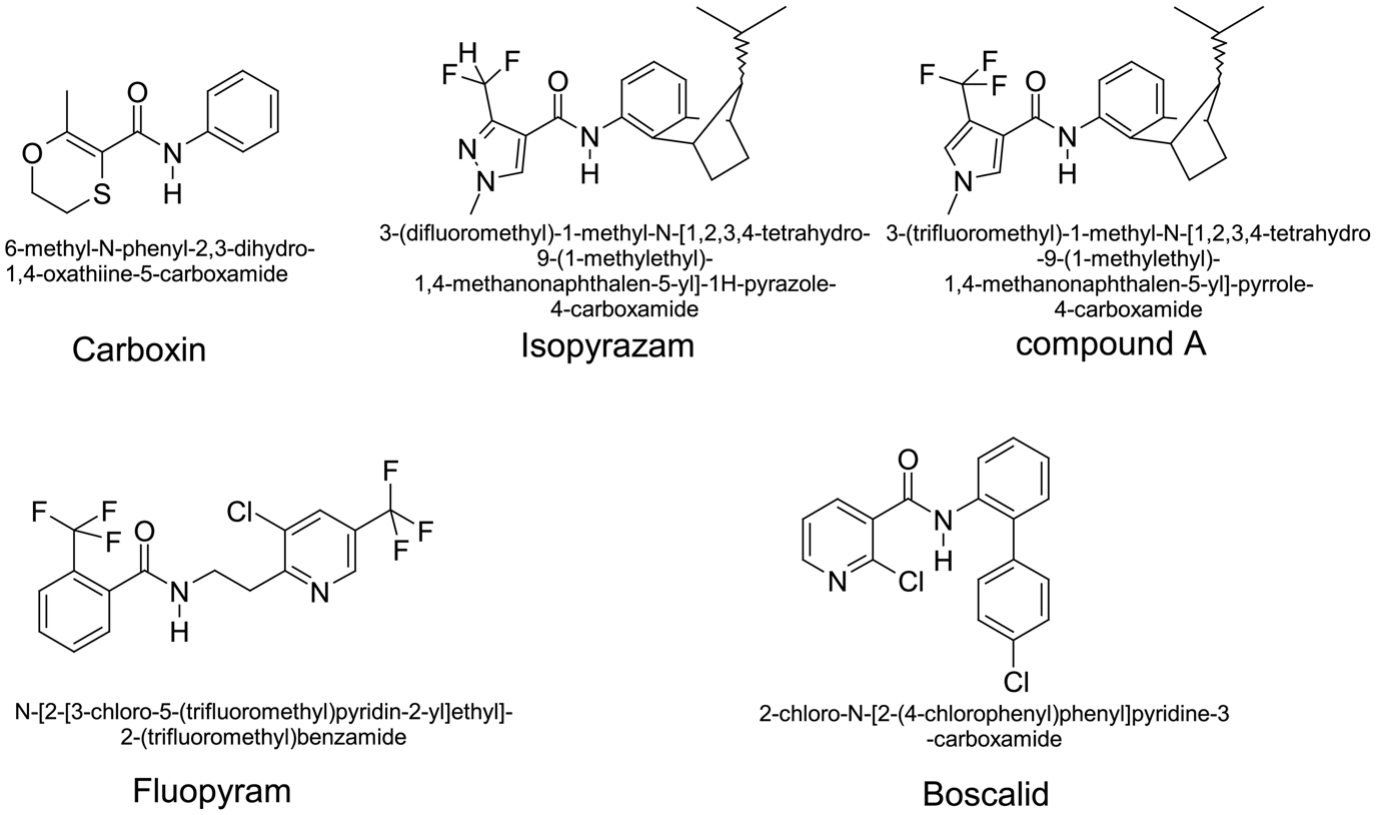
Chemical structure of carboxamides used in the study, trivial and IUPAC denominations. The generic structure of carboxamides can be divided into 3 main parts. (i) the “acid core” which differs from its ring structure: oxathiin (Carboxin), pyrazole (Isopyrazam), pyrrole (compound A), pyridine (Boscalid), substituted benzene (Fluopyram) (ii) the “linker” which is composed by a benzene ring or a 2-carbon aliphatic spacer in Fluopyram, (iii) the bulky hydrophobic rest, missing in Carboxin.The aim of the figure legend should be to describe the key messages of the figure, but the figure should also be discussed in the text. An enlarged version of the figure and its full legend will often be viewed in a separate window online, and it should be possible for a reader to understand the figure without switching back and forth between this window and the relevant parts of the text. Each legend should have a concise title of no more than 15 words. The legend itself should be succinct, while still explaining all symbols and abbreviations. Avoid lengthy descriptions of methods.

Compounds used for selection following UV mutagenesis were chosen both for structural diversity and relevance to the crop protection market (Fig. 1).

Two different UV incidences were used for most selection compounds 75 mJ cm^−2^ or 50 mJ cm^−2^ leading to 80% and 50% lethality after UV treatment respectively. Frequency of resistance development ranged between 7.2 E^−7^ for Carboxin at the minimal selective concentration (MSC, see material and methods) to the absence of growing colonies for Fluopyram at 5×MSC (Table 1). The strength of UV treatment had a minor impact on resistance frequency suggesting that mutation rate, increasing with UV exposure and percentage of survival after UV treatment, decreasing with UV exposure, is finely balanced in this range of survival rates. For all compounds, except Boscalid and compound A, a pyrrole analogue of Isopyrazam, a decrease in the frequency of growing colonies was observed with 5×MSC selection which is consistent with higher concentrations of the active ingredient providing more stringent selection conditions. With Boscalid a higher frequency of resistance was observed with the 5×MSC selection. However, careful examination of the AE agar plates supplemented with 150 µM Boscalid (5×MSC) displayed white precipitate which developed over time. Taken together this suggests that the Boscalid was precipitating in the media and that the concentration of biologically available Boscalid at the time of selection was lower in the Boscalid 150 µM supplemented plates compared to the ones supplemented with Boscalid at 30 µM. No significant change in the frequency of resistant strain development could be observed between MSC and 2.5× MSC with the pyrrole carboxamide compound A.

**Table 1:** Mutagenesis experiments overview.

Selection	UV treatment	Plated cells (10^6^)	Collected colonies	Frequency
Carboxin 85 µM (MSC)	50	90	56	6.2E-07
Carboxin 85 µM (MSC)	75	90	65	7.2E-07
Carboxin 425 µM (5× MSC)	50	90	1	1.1E-08
Carboxin 425 µM (5× MSC)	75	90	1	1.1E-08
Isopyrazam 3 µM (MSC)	50	90	29	3.2E-07
Isopyrazam 3 µM (MSC)	75	90	37	4.1E-07
Isopyrazam 15 µM (5× MSC)	50	90	13	1.4E-07
Isopyrazam 15 µM (5× MSC)	75	90	15	1.7E-07
compound A 100 µM (MSC)	50	90	13	1.4E-07
compound A 100 µM (MSC)	75	90	19	2.1E-07
compound A 250 µM (2.5× MSC)	50	90	19	2.1E-07
compound A 250 µM (2.5× MSC)	75	90	17	1.9E-07
Fluopyram 25 µM (MSC)	50	90	64	7.1E-07
Fluopyram 25 µM (MSC)	75	90	67	6.3E-07
Fluopyram 125 µM (5× MSC)	50	90	0	nd
Fluopyram 125 µM (5× MSC)	75	90	2	2.2E-08
Boscalid 30 µM (MSC)	50	50	64	1.3E-06
Boscalid 150 µM (5× MSC)	50	1.6	14	8.8E-06

### Molecular characterization of carboxamide selected mutants

Following primary selection, 482 colonies were picked from the primary selection plates (Table 1) and re-isolated on the same selective media. Following this process, 30 strains unable to grow consistently were identified as false positives (Table 2). All other strains were cultured and sequence analysis of the *MgSDHB*, *MgSDHC* and *MgSDHD* genes was undertaken. Mutations leading to amino acid substitutions in the target proteins were detected in 444 out of 452 of the selected strains (98.2%). Target mutations were identified in all three Qp site encoding subunits and as many as 27 different substitution types were identified. Rare cases of double substitutions were observed in SDHB (Boscalid selection), SDHC (Isopyrazam selection) and SDHD (Carboxin selection). However, no mutants carrying substitutions in more than one subunit simultaneously were obtained. The absence of target mutation was only observed for 8 strains (1.8%). Additional controls performed on these strains including repeated isolation under selective conditions and re-sequencing of the four SDH encoding genes confirmed non target site resistance mechanisms can be selected under low compound selection pressure.

**Table 2:** Selected colonies and detailed quantitative and qualitative overview of the SDH substitutions selected with different carboxamides concentrations.

Compound		Carboxin		Isopyrazam		compound A		Fluopyram		Boscalid	
Concentration for selection		MSC	5× MSC	MSC	5× MSC	MSC	2.5× MSC	MSC	5× MSC	MSC	5× MSC
False positives		12	-	9	-	6	-	3	-	-	-
True positives		109	2	63	22	33	29	128	2	50	14
No SDH substitution		3	-	-	-	5	-	-	-	-	-
With SDH substitution		**106**	**2**	**63**	**22**	**28**	**29**	**128**	**2**	**50**	**14**
Codons	Substitution										
tct>ttt	SDHB_S218F	-	-	-	-	-	-	1	-	-	-
cca>aca	SDHB_P220T	-	-	-	-	-	-	1	-	-	-
cca>cta	SDHB_P220L	-	-	-	-	-	-	2	-	-	-
tcc>ccc	SDHB_S221P	2	-	-	-	-	-	-	-	-	-
aac>cac	SDHB_N225H	1	-	-	-	-	-	-	-	-	-
aac>atc	SDHB_N225I	-	-	-	-	-	-	1	-	-	-
cga>cca	SDHB_R265P	4	-	-	-	-	-	-	-	-	-
cac>cta/ctc/ctt	SDHB_H267L	7	2	16	3	2	-	7	-	6	-
cac>aac	SDHB_H267N	11	-	-	-	-	-	-	-	-	-
cac>cag	SDHB_H267Q	-	-	-	-	-	-	-	-	-	2
cac>tac	SDHB_H267Y	61	-	-	-	-	-	-	-	38	5
att>gtt	SDHB_I269V	11	-	-	-	-	-	-	-	-	-
aac>aag	SDHB_N271K	-	-	-	-	-	-	6	-	-	-
acc>atc	SDHC_T79I	-	-	-	-	-	-	-	-	-	4
tcg>ggg	SDHC_S83G	-	-	1	-	-	-	4	2	-	-
gcc>gtc/gtt	SDHC_A84V	-	-	-	-	-	-	73	-	-	-
gcc>atc	SDHC_A84I	-	-	-	-	-	-	1	-	-	-
ctc>ccc	SDHC_L85P	2	-	-	-	-	-	-	-	-	-
acc>aag/aaa	SDHC_N86K	-	-	8	3	4	9	6	-	-	1
cgc>tgc	SDHC_R87C	-	-	-	-	-	-	24	-	-	-
gac>gtc	SDHC_V88D	-	-	-	-	1	-	-	-	-	-
cac>cgt	SDHC_H145R	1	-	-	-	-	-	-	-	-	-
cat>cgt/cga	SDHC_H152R	-	-	37	16	21	20	-	-	-	1
gac>gag/gaa	SDHD_D129E	2	-	-	-	-	-	-	-	2	-
gac>ggc	SDHD_D129G	2	-	1	-	-	-	-	-	1	1
gac>tcc	SDHD_D129S	1	-	-	-	-	-	-	-	-	-
gac>acc	SDHD_D129T	-	-	-	-	-	-	1	-	-	-
	Multiple SDHB	-	-	-	-	-	-	1	-	-	-
	Multiple SDHC	-	-	-	-	-	-	-	-	3	-
	Multiple SDHD	1	-	-	-	-	-	-	-	-	-

Numbers correspond to the total number of colonies belonging to each sub-group (true/false positives and with/without mutation in the SDH encoding genes) for each selection condition (See material and methods). The second part details the number of colonies for the 27 SDH substitution type selected on each carboxamide concentration.

Selected substitution types appeared to be both compound and concentration dependent. Interestingly, clear decreases in resistance frequencies were observed for Fluopyram and Carboxin upon shifting selection from MSC to 5× MSC (Table 1). These were accompanied by a drastic reduction in the diversity of the different substitution types (Table 2). For Fluopyram the diversity of selected substitution types decreased from 13 at MSC to 1 at 5×MSC. The most frequent substitution at the MSC (SDHC_A84V, 57%) was not observed at 5×MSC. On the other hand SDHC_S83G substitution representing only 3.1% of the mutants at MSC was the only substitution type observed at 5× MSC. Similarly, Carboxin selected 13 substitutions at MSC but only the SDHB_H267L substitution could be found at 5×MSC. At the MSC, the most frequent substitution conferring Carboxin resistance was SDHB_H267Y accounting for 57.5% of the mutants, while SDHB_H267L represented only 6.6% of the mutants under these conditions. These results suggest that a diverse range of substitutions can lead to resistance to low levels of Fluopyram and Carboxin but at the more stringent 5× MSC selection substitution types leading to lower resistance factors are eliminated. Although less marked, diversity of substitution types was also reduced from 5 to 3 and from 5 to 2 with increased Isopyrazam and compound A selection respectively. When comparing the substitution types obtained across the different carboxamides, the substitution patterns were most similar for Isopyrazam and its pyrrole homologue. For these 2 molecules, the substitutions SDHB_H267L, SDHC_N86K and SDHC_H152R were the most frequently isolated. Given the similarity in the structures of these two carboxamides, similar substitution pattern might be expected, however the SDHB_H267L substitution was found much more frequently with Isopyrazam suggesting subtle differences in the molecular recognition of those two compounds by the enzyme. Seven of the isolated substitution types were found in multiple selection conditions, most notably the SDHB_H267L substitution which was selected with all compounds tested (Table 2). Interestingly, other substitutions were unique to the individual compounds used for selection, this was the case for many mutation types selected with Carboxin (8 out of 12) and with Fluopyram (9 out of 12). These different substitution patterns suggest subtle differences in the way the different carboxamide compounds interact with SDH.

### Localization of amino acid substitutions in the primary structure of SDH proteins

**Figure 2:**
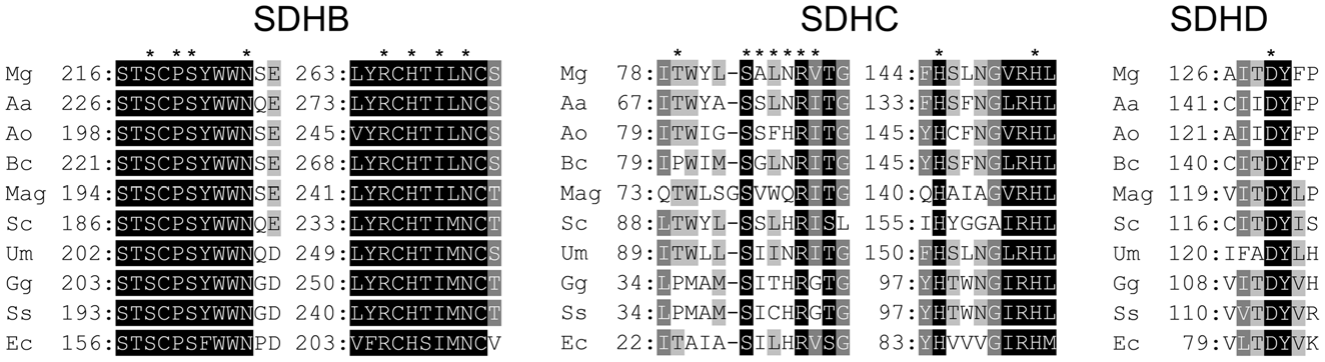
Localization of substituted amino acids in the SDH subunits of *M. graminicola* UV mutants and degree of conservation across species. Asterix indicate substituted residues, conserved residues are shaded in black, dark grey and light grey corresponding to 100%, 80% and 60% conservation respectively. Sequences are from *M. graminicola* (Mg), *Alternaria alternata* (Aa), *Alternaria oryzae* (Ao), *B. cinerea* (Bc), *Magnaporthe grisea* (Mag), *S. cerevisiae* (Sc), *Ustilago maydis* (Um), *G. gallus* (Gg), *S. scrofa* (Ss) and *E. coli* (Ec).

Among the 3 subunits composing the Qp site, SDHB is by far the most conserved, displaying 57% identity across ascomycetes sequences used in the alignment (Fig. 2). By contrast SDHC and SDHD display rather poor primary sequence conservation with 22% and 21% identity respectively across the ascomycetes species used in the alignment.

Our mutagenesis screen for carboxamide resistance led to the identification of 18 substituted amino acids, among which 13 were affecting highly conserved residues of the Qp site. A great proportion of these residues has been shown to be functionally involved in ubiquinone binding and reduction catalysis in other species [13], [38], [39], [40], [41].

Two regions prone to substitution could be identified on SDHB, one between amino acids S218 and P225 and the other between amino acids R265 and N271. All these SDHB amino acid positions are strictly conserved across species. On SDHC, one major region prone to amino acid substitution was identified between amino acid T79 and V88, with a continuous stretch of substituted positions between S83 and V88. This later contains the S83 and R87 highly conserved Qp site residues and four neighbouring positions which are not conserved across species (A84, L85, N86 and V88). The nature of the residues present at these positions may have an influence on Qp site structure and properties and thus, on the biological profile of carboxamides. Finally, the H145 and H152 histidines, which are also strictly conserved across species, were both found substituted with arginines. On SDHD, D129 was the only substituted position; together with its neighbouring Y130 these 2 positions represent the only conserved residues across the whole protein in the SDHD alignment.

### Cellular and mitochondrial sensitivity of resistant mutants to carboxamides

To explore the sensitivity profiles conferred by amino acid substitutions at the cellular and mitochondrial level, we determined sensitivity towards 4 different compounds for 26 of the identified substitution types both *in vivo* using liquid culture growth (AE media) and *in vitro* using purified mitochondria and the succinate: Q_0_/DCPIP activity test (Table 3). To ensure that only target site resistance was studied, a series of comparative tests with strains carrying similar target mutations was performed and only strains that displayed consistent resistance profiles were selected for further study. One representative strain was selected for all substitution types apart from SDHB_S221P which seemed to result in a significant fitness penalty as the two strains carrying this substitution displayed very significant growth defects. All other strains displayed similar levels of growth in liquid AE media. Resistance factors (RF) were determined as the ratio IC_50_mutant/IC_50_WT. The values of *in vivo* resistance factors for strains selected on each compound ranged between the following values: 4.4 (SDHC_H145R) to 39.7 (SDHD_D129S) for Carboxin selection, 22.9 (SDHD_D129G) to 464.4 (SDHC_N86K) for Isopyrazam selection, 5 (SDHB_P220L) to 126.8 (SDHC_S83G) for Fluopyram and 21.7 (SDHB_H267Q) to 564.3 (SDHC_S83G) for Boscalid. Because of the very poor succinate: Q_0_/DCPIP activity displayed by some mutants *in vitro*, we calibrated our test by adjusting enzyme concentrations at a similar initial velocity (see material and methods). As IC50s are dependent on amount of enzyme used in the test, the ranking of RFs obtained *in vivo* compared to the *in vitro* values can differ notably. For example the SDHD_D129T substitution which displayed an *in vitro* RF of 4095 towards Isopyrazam only ranks as the fifth strongest mutation *in vivo* (RF: 33). Similar discrepancies between the *in vivo* and *in vitro* rankings can be observed for all of the carboxamides tested. However, these data enable a comparison of the differential behaviours displayed towards the 4 carboxamides tested by a given mutated enzyme. Qp site mutations are well known to influence SDH enzyme activity and stability upon catalysis, [13], [38], [41]. Discrepancies observed between the *in vivo* and *in vitro* sensitivity data suggested that the impact of substitutions on enzymatic activity needed to be addressed to have a fuller understanding of the factors influencing the resistance displayed *in vivo*.

**Table 3:** *In vivo* and *in vitro* IC50s and resistance factors overview.

		IC50 (nM)							
		Carboxin		Isopyrazam		Fluopyram		Boscalid	
		*in vivo*	*in vitro*	*in vivo*	*in vitro*	*in vivo*	*in vitro*	*in vivo*	*in vitro*
WT (IPO323)		6470 ±1085	1156 ±102	55 ±2	5 ±0.3	584 ±100	44 ±2.2	455 ±47	60 ±1.8

		Resistance Factors (RFs)							
		Carboxin		Isopyrazam		Fluopyram		Boscalid	
Substitution	Strain	*in vivo*	*in vitro*	*in vivo*	*in vitro*	*in vivo*	*in vitro*	*in vivo*	*in vitro*
None	IPO323	1	1	1	1	1	1	1	1
B_S218F	Flu41	5.3	46.4	15.0	18.2	18.0	276.5	6.5	46.3
B_P220T	Flu110	0.1	0.7	3.8	1.8	11.5	84.9	6.1	28.3
B_P220L	Flu75	1.4	4.4	4.8	1.2	5.0	3.3	2.2	2.6
B_N225H	Cbx106	20.2	38.0	13.6	15.7	8.0	15.2	12.2	20.1
B_N225I	Flu7	10.2	6.9	15.0	7.0	17.5	25.0	12.9	18.5
B_R265P	Cbx56	20.1	271.1	7.6	88.3	2.8	63.0	13.3	256.0
B_H267L	Cbx29	14.5	1959.3	152.2	3248.5	22.4	766.1	258.0	9126.6
B_H267N	Cbx96	12.6	232.7	4.5	11.1	9.9	68.3	3.4	34.8
B_H267Q	Bos59	4.4	85.5	12.5	34.6	0.9	11.8	21.7	1034.3
B_H267Y	Cbx28	14.1	65.9	16.1	142.6	0.6	5.1	305.6	473.8
B_I269V	Cbx1	12.4	19.9	5.0	3.6	10.0	18.0	4.2	12.7
B_N271K	Flu33	2.6	1069.2	5.7	89.5	10.0	2481.0	3.4	223.7
C_T79I	Bos64	5.2	62.0	15.8	56.4	2.2	12.3	18.9	145.6
C_S83G	Flu1	10.7	11.7	61.2	38.6	126.8	201.0	564.3	1392.4
C_A84V	Flu4	0.8	1.2	3.5	3.1	19.0	102.7	2.6	3.7
C_A84I	Flu21	1.2	1.8	8.8	8.1	31.9	260.0	2.9	4.4
C_L85P	Cbx12	11.5	265.4	10.0	35.3	5.8	130.1	4.1	34.7
C_N86K	Izm7	23.8	135.9	464.4	849.9	49.9	245.8	78.1	160.2
C_R87C	Flu9	3.2	nd*	3.7	nd*	6.1	nd*	18.8	nd*
C_V88D	Ol50	4.4	67.2	36.5	140.4	5.9	55.4	5.0	59.8
C_H145R	Cbx76	4.4	nd*	27.6	nd*	2.0	nd*	17.3	nd*
C_H152R	Izm1	13.2	45.4	133.9	95.0	11.5	14.3	30.2	58.1
D_D129E	Cbx22	35.9	631.6	6.8	145.3	1.3	10.5	42.8	1220.9
D_D129G	Cbx67	17.2	2030.3	22.9	583.2	5.6	1880.2	37.1	2052.4
D_D129S	Cbx101	39.7	377.7	10.8	82.5	1.9	12.3	15.9	341.2
D_D129T	Flu29	60.4	FR**	33.5	4095.5	11.5	3724.4	78.3	5515.8

Upper panel: *In vivo* and *in vitro* IC50 values obtained for the WT (IPO323) ±S.E. (triplicate). Lower panel: resistance factors (RFs) based on IC50 assessment for a selected subset of representative strains. Presented values are based on the ratio of the means of three individual determinations for the *in vivo* values and based on the ratio of a single determination for the *in vitro* values. Presented *in vitro* values were obtained by calibrating mitochondrial dilutions to obtain similar initial velocity (see material and methods). Cbx strains were originally obtained on Carboxin selection media, Flu on Fluopyram, Bos on Boscalid, Izm on Isopyrazam, Ol on pyrrole compound A. nd*: IC_50_ could not be determined because fitted curves were not tending to 100% inhibition at infinite AI concentration. FR** (full resistance), no sufficient inhibition detected at highest inhibitor concentration tested.

### Carboxamide pharmacophore elucidation using available structural information

**Figure 3:**
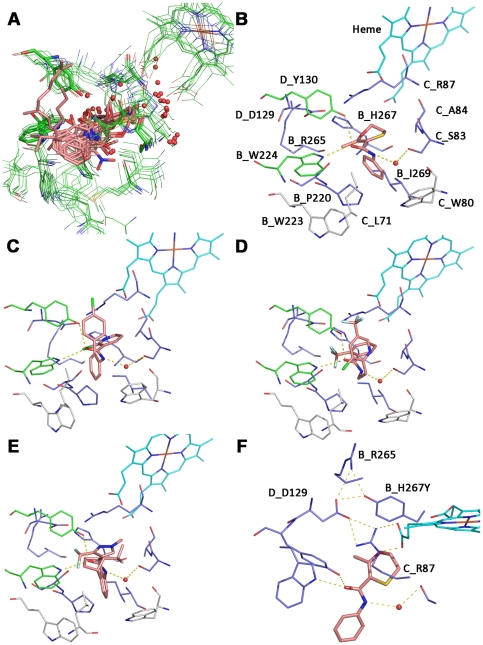
Tridimensional model of the *M. graminicola* SDH with Qp site docked carboxamides and interactions to substituted residues. A: Superposition of all complex II crystal structures with a resolution higher than 3 Å. Only amino acids that are in a 5 Å radius to the bound ligands in the quinone binding site are shown. Amino acids and the heme are represented in lines, the ligands in sticks and water molecules as non bonded spheres. The color code is presented according to atom types. Amino acid and heme carbons are colored in green, ligands carbons in salmon. B: putative binding mode of carboxin in a tridimensional model of *M.graminicola* SDH. The heme carbons are represented in cyan sticks, the carboxin carbon atoms in salmon. Amino acids that are involved in resistance after mutation are colored in dark blue, amino acids that make key interactions are shown in green and amino acids that are in close proximity to the ligand but which were not found substituted in this study are colored in grey. Hydrogen bonds are shown as yellow dotted lines. C: Putative binding mode of Boscalid in *M.graminicola* SDH. D: Putative binding mode of Fluopyram in *M. graminicola* SDH. E: Putative binding mode of Isopyrazam in *M. graminicola* SDH. F: Model of *M. graminicola* SDH where SDHB histidine 267 is mutated into a tyrosine. The putative binding mode of the mutated enzyme with Carboxin is shown.

In order to obtain a clearer understanding of the structural basis for the differential resistance profiles observed across carboxamides in the SDH mutants, a prediction of the binding modes for the carboxamides used in this study was required. In order to generate putative binding modes, all publicly available crystal structures were analysed in a first step to identify key pharmacophoric features (see material and methods). All crystal structure ligands are equipped with a hydroxyl or carbonyl group making hydrogen bonds to B_Trp164 and D_Tyr83 (amino acid numbering according to *E. coli* SQR). In the crystal structure 1NEN the quinone ring is sandwiched by B_Pro160 and C_Ile28 [14]. The overlay of all structures onto each other shows that some ligands are not superimposing exactly on the quinone ring but all ligands display a scaffold that lie in the same plane (Fig. 3A). This was partly described already by Horsefield *et al.* who divided the binding pocket into a Q1 and Q2 sites [10]. The ligands of the structures with PDB codes 1NEK, 1NEN, and 2ACZ are equipped with lipophilic alkyl groups making hydrophobic interactions at the entrance of the quinone pocket. The alkyl groups are oriented perpendicular relative to the quinone plane (Fig. 3A). In carboxin SQR co-crystal structures, the orientation of the methyl group of the oxathiin ring was a matter of controversy. Initially it was proposed that the methyl group is oriented towards C_Ser27 (2FBW) [15] but more recent crystal structure elucidations with both chicken (2WQY) and *E. coli* SQR (2WDQ) enzymes demonstrated a 180° flipped oxathiin ring [40]. Moreover, recent structures elucidated with SQR-ubiquinone binding site inhibitors further confirm this trend. The structure 3AE4 shows that the Br atom, displaying a well defined electron density, has the same orientation as the methyl in the oxathiin ring of carboxin. In other structures the CF3, or methyl group were consistently built on the same side (e.g. PDB code 3ABV, and 3AE5). Mutagenesis and kinetic data have demonstrated the importance of the conserved SDHC Qp site serine (SDHC_S83 in *M.graminicola*, C_Ser27 in *E.coli*) in ubiquinone binding and catalysis [13]. In addition, there are two crystal structures with quinone scaffolds where a hydrogen bond between the ligands and C_Ser27 could be observed (1YQ3, 1YQ4) [42]. This residue is proposed to be a key residue interacting with ubiquinone during its proposed movement within the Qp site during catalysis [10]. Based on the high structural similarity to the carboxamides described in this study, the interaction observed in the chicken Carboxin-SQR co-crystal structure 2WQY gives a valuable indication of the binding mode of carboxamides. In this crystal structure a hydrogen bond mediated by a water molecule was observed between the ligand amide NH and the C_Ser27 hydroxyl [15].

### Inhibitor docking studies in a *M. graminicola* SDH homology model

Utilizing observed protein ligand interactions, putative binding modes were generated for Boscalid, Isopyrazam, and Fluopyram (Fig. 3C–E). All three fungicides share similar key interactions displaying hydrogen bonds to SDHB_W224 and SDHD_Y130 through the amide oxygen and the hydrogen bond of the amide NH to SDHC_S83 mediated by a water molecule. The involvement of the SDHC_S83 in the establishment of a hydrogen bond upon carboxamide binding is supported experimentally by the high resistance factors (up to 564 fold *in vivo*) observed across all ligands with the SDHC_S83G mutant. The acid part of all carboxamides (oxathiin, pyrazole, phenyl and pyridine) is sandwiched between C_Arg87 and B_Ile269 and form Van-der-Waals interactions to SDHB_I269, and cation-π interaction to SDHC_R87. The SDHC_R87C mutation abolishes the cation-π interaction; *in vivo*, this mutation conferred only limited carboxamide sensitivity decrease (RFs between 3.2 and 18.8). *In vitro*, the effect of the SDHC_R87C substitution on enzyme inhibition looked more complex as only partial inhibition could be reached at maximal concentration of the inhibitor despite of an otherwise WT like response. The SDHB_I269V mutation also conferred limited resistance factors (ranging between 4 to 20 fold *in vitro*). A valine can still compensate for some of the lipophilic interactions carried out by SDHB_I269. The ortho substituents of the acid parts (methyl in Carboxin, CF2 in Isopyrazam, CF3 in Fluopyram, and Cl in Boscalid) are pointing towards SDHB_S221 which corresponds to the orientation suggested by Ruprecht *et al* [40].

SDHB_R265 and SDHD_D129 are not involved in direct interactions to the ligands according to the binding models but form a hydrogen bond network with the heme and the imidazole ring of SDHB_H267. SDHB_H267 is positioned at the bottom of the Qp site. Depending on the organism this residue either makes a direct hydrogen bond to SDHD_D129 (observed for all *G. gallus* and *S. scrofa* SQR crystal structures) or to one heme propionate (observed for all *E. coli* SQR structures). By analyzing the electron density for the histidine sidechain it was suggested that this amino acid can adopt multiple conformations [10]. In our proposed binding modes for *M. graminicola* SDH, SDHB_H267 makes a direct hydrogen bond to SDHD_D129 and edge to face stacking to the four different inhibitors. However, an alternative binding mode can be hypothesized, where a direct hydrogen bond of the histidine to the pyridine of Boscalid, to the pyrazole of Isopyrazam, and to the oxathiin of Carboxin occurs. In this case SDHB_H267 cannot make direct hydrogen bonds to SDHD_D129 or one heme propionate anymore. This alternative is supported by the recently solved crystal structure of the *E. coli* SQR enzyme containing the histidine to threonine substitution (PDB code 2WP9). In this study, the complex II was shown to remain stable and active in a case where neither a hydrogen bond to the heme propionate nor to SDHD_D129 are formed [43]. A mutation of B_H267Y leads to high *in vitro* resistance factors for Boscalid (RF = 473.8), Isopyrazam (RF = 142.6), and Carboxin (RF = 65.9). In order to understand this in more detail a model for *M. graminicola* SQR with the SDHB_H267Y substitution was generated (Fig. 3F). The aromatic sidechain makes edge to face stacking to the inhibitors and the tyrosine hydroxyl group a hydrogen bond to SDHD_D129. In this configuration, a direct hydrogen bond of the tyrosine to the hydrogen bond accepting groups of Boscalid, Isopyrazam, and Carboxin is unlikely potentially impairing the binding of these molecules and thereby explaining the higher resistance factors observed for these molecules. Moreover, this model would predict the low resistance towards Fluopyram which is not equipped with an acceptor group and Carboxin with the second lowest resistance factor (*in vivo* RF = 14.1) is equipped with the weakest accepting group followed by Isopyrazam and Boscalid.

SDHB_P220 is a fully conserved Qp site residue sandwiching the quinone ring together with a residue corresponding to SDHC_A84 in all ubiquinone-SDH co-crystal structures [14], [15], [16]. In the binding models, the proline displays hydrophobic interactions with the amine part of the carboxamides (aliphatic linker in Fluopyram; phenyl group in Carboxin, Boscalid, and Isopyrazam). *In vitro*, resistance factors are in a single digit range except for Fluopyram (RF = 85) and Boscalid (RF = 28) with the SDHB_P220T mutant. SDHC_A84 is predicted to interact via Van-der-Waals forces with the aliphatic linker of Fluopyram and the pyridine ring. This aliphatic linker is a specific feature of Fluopyram compared to the other carboxamides analysed in this study. The linker is in the z-dimension sterically more demanding and is getting closer to SDHC_A84 compared to phenyl ring systems of Carboxin, Boscalid, and Isopyrazam. In agreement with these predictions, Fluopyram binding is found to be specifically affected by the SDHC_A84V/I substitutions as shown by over 30 fold higher RFs *in vitro* compared to the phenyl ring carboxamides. Furthermore, higher resistance factors are observed with the bulkier amino acids substitution, SDHC_A84I which is in line with the proposed interaction. Interestingly, the amino acid at SDHC_A84 position is not conserved across fungal species and may contribute to some of the biological profile observed for these carboxamide subclasses.

The SDHB_W223, SDHC_L71, and SDHC_W80 residues, are all in Van-der-Waals distance to the amine part of all ligands. However, no substitutions at these positions were observed in this study. The substituted residues, SDHB_S218, SDHB_N225, SDHB_N271; SDHC_T79, SDHC_L85, SDHC_N86, SDHC_V88, SDHC_H145, and SDHC_H152 are not predicted to directly interact with carboxamides but amino acid substitutions more distant from the binding pocket may influence the shape and electronics of the Qp site and therefore have a negative impact on ligand binding. A putative long-range effect and/or a rearrangement of a large zone affecting part of the Qp site is particularly likely for the SDHC_H145 position which was substituted by an arginine upon Carboxin selection as this is the furthest substituted residue from the Qp site. Despite a distance of ∼10 Å to the closest atom of docked Carboxin, the SDHC_H145 is a ligand of the heme and therefore may influence the Qp site structure and/or properties. According to available SDH crystal structures, the propionate side chains of the heme can be considered part of the Qp site. Furthermore, it was shown that the heme itself directly participates in electron transfer during catalysis [11]. In yeast and in *E. coli*, modification of the coordinated histidine impairs ubiquinone binding and catalysis efficiency of the SDH enzyme [39], [42], [44]. Therefore, the SDHC_H145R substitution in *M. graminicola* may influence heme properties and/or positioning which in turn might have a negative impact on inhibitor binding.

SDHC_N86K and SDHC_H152R were the most frequent substitutions upon Isopyrazam and compound A selection. Both were also selected by Boscalid but less frequently and the SDHC_N86K substitution was also found upon Fluopyram selection. In the *M. graminicola* tridimensional SDH model, these residues are not predicted to directly interact with the inhibitor yet are in the close vicinity to the heme, again making a direct influence on heme positioning possible.

### Enzyme efficiency determination

To compare specific activities conferred by the different amino acid substitutions we first determined the level of SDH enzyme in mitochondrial preparations for each of the resistant strains (Table 4). SDH enzyme carries a covalently bound FAD at the succinate oxidation site located within the SDHA subunit [45], this feature enables the accurate quantification of the SDH enzyme even in complex samples [46]. Overall, covalent FAD values differed at most by a factor 4 between samples, suggesting that the strains carrying less active SDHs are not compensating for this effect by overexpression of the enzyme. This result was further validated by western blot using anti *M. graminicola* SDHB antibodies (data not shown).

**Table 4:** Enzymatic efficiencies determinations for selected subset of strains representative of each mutation type.

Mutation	Strain	Covalent FAD^a^	MTT/PMS activity^b^			Q_0_/DCPIP activity^c^		
none	IPO323	330.3±23.6	474	±62	(100%)	484.4154407	±42	(100%)
B_S218F	Flu41	344.4±14	416	±67	(88%)	92.87292759	±18	(19%)
B_P220T	Flu110	378.0±4.7	720	±110	(152%)	107.7682646	±9	(22%)
B_P220L	Flu75	304.5±5.8	717	±84	(151%)	231.8025324	±16	(48%)
B_N225H	Cbx106	299.5±3.8	850	±175	(179%)	334.8801055	±33	(69%)
B_N225I	Flu7	281.0±14.2	651	±98	(137%)	188.7997691	±19	(39%)
B_R265P	Cbx56	363.1±19.7	217	±53	(46%)	41.7782216	±10	(9%)
B_H267L	Cbx29	519.5±10.4	381	±37	(80%)	64.57824113	±7	(13%)
B_H267N	Cbx96	246.7±4.3	553	±77	(117%)	94.85037408	±15	(20%)
B_H267Q	Bos59	453.8±8.5	334	±40	(70%)	65.25108944	±6	(13%)
B_H267Y	Cbx28	438.4±25.5	190	±44	(40%)	45.65897328	±18	(9%)
B_I269V	Cbx1	387.2±21.0	545	±147	(115%)	416.7898614	±73	(86%)
B_N271K	Flu33	286.9±7.8	218	±26	(46%)	25.98331188	±5	(5%)
C_T79I	Bos64	241.9±3.0	501	±70	(106%)	124.887427	±16	(26%)
C_S83G	Flu1	368.6±12.4	714	±97	(151%)	286.9379225	±34	(59%)
C_A84V	Flu4	229.6±9.1	850	±119	(179%)	419.0858506	±59	(87%)
C_A84I	Flu21	631.5±15.4	596	±95	(126%)	245.6437942	±32	(51%)
C_L85P	Cbx12	320.8±24.9	334	±56	(70%)	143.5533934	±28	(30%)
C_N86K	Izm7	284.7±6.6	407	±86	(86%)	200.6388817	±35	(41%)
C_R87C	Flu9	333.5±9.3	389	±46	(82%)	117.638248	±12	(24%)
C_V88D	Ol50	200.5±7.8	838	±170	(177%)	139.7192089	±21	(29%)
C_H145R	Cbx76	377.8±27.2	555	±98	(117%)	97.84219544	±12	(20%)
C_H152R	Izm1	156.7±8.8	227	±34	(48%)	106.7682223	±22	(22%)
D_D129E	Cbx22	211.4±7.6	491	±123	(104%)	202.5551707	±30	(42%)
D_D129G	Cbx67	180.9±3.8	808	±80	(171%)	91.9077442	±9	(19%)
D_D129S	Cbx101	453.8±8.5	534	±84	(113%)	111.2427749	±17	(23%)
D_D129T	Flu29	253.9±10.5	568	±69	(120%)	29.33687605	±6	(6%)

a Covalent FAD is expressed in pmol covalend FAD mg of protein^−1^ and is an estimation of the total amount of succinate dehydrogenase enzyme present in the mitochondrial preparation, presented values are the average of four individual determinations ± S.D.

b MTT/PMS activity expressed as µmol PMS reduced MTT min^−1^ µmol covalent FAD^−1^. Values represent the means of at least 10 determinations ± S.D.

c Qo mediated DCPIP reduction expressed in µmol reduced DCPIP min^−1^ µmol covalent FAD^−1^ in the presence of 1 mM Qo. Values represent the means of at least 10 determinations ± S.D.

The malonate sensitive succinate: PMS/MTT activity test is classically considered as a measurement of the SDHA-B dimer [47]. This activity does not require the functional reduction of the ubiquinone at the Qp site and was accordingly not affected upon carboxamide addition in *M. graminicola* [48]. Activity levels varied greatly among mutants, ranging between 46% and 179% of the WT enzyme activity. A wide range of effects could be observed even when substitions affected similar position as observed with the SDHB_H267Y and SDHB_H267N which displayed succinate: PMS/MTT activities of 40% and 117% of the WT activity respectively. Interestingly, mutations on SDHC and SDHD also have a major impact on this activity. As the PMS electron donor site has not been identified yet, we suspect that electron distribution within the enzyme might be affected in our SDH mutants, which could in turn favour or disfavour reduction of this substrate at its reduction site(s) [43], [47], [49]. Similar variations have also been reported for other Qp site mutations in other studies [41], [50].

*In vivo*, the electrons derived from succinate have to be transferred to its acceptor (ubiquinone) to enable the enzymatic oxidizing of novel molecules of succinate. The succinate: Qo/DCPIP activity is a measure of the succinate: ubiquinone reductase activity, which is the most relevant one biologically. *In vitro*, full inhibition of the WT enzyme can be reached using all four carboxamides compared in this test.

All mutants displayed weaker ubiquinone reductase activity compared to the wild type. The weakest effect was detected for the SDHC_A84V mutant which was 87% as active as the WT. The strongest impairment was displayed by the SDHB_N271K mutant with only 5% residual activity. As might be expected, different substitutions at the same residue can result in differential impact on enzyme efficiency. This effect seems to be linked to the degree of steric or physico chemical conservation displayed by the substitutive amino acid. For example, the SDHD_D129E conservative substitution maintains 42% of WT activity whiles the non conservative SDHD_D129G/S/T substitutions impact enzyme activity much more strongly (from 6 to 23% remaining activity). The same observation can be made for the SDHC_A84V variant which is more active (87%) than the SDHC_A84I (51%) counterpart which carries a larger substituent.

**Figure 4:**
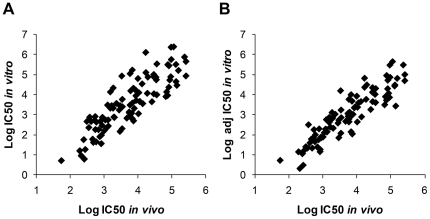
Scatter plot presenting *in vivo* LogIC_50_ (nM) versus *in vitro* LogIC_50_ (nM) adjusted (B) or not (A) for enzyme efficiency. All data extracted from table 3 and adjusted for amount of enzyme used in the sensitivity test in panel B. Adjustment was performed using enzyme efficiency data extracted from table 4 following a simple equation: if percent efficiency is denoted by E then the efficiency adjusted IC_50_ = observed IC_50_×E/100. Following this adjustment the correlation was improved for all compounds.

The straight comparison of the *in vivo* log IC_50_ estimates and *in vitro* log IC_50_ estimates across the different strains for any given compound displayed reasonable correlation for each of the four compounds considered here (Fig. 4A). We attempted to correct IC_50_ values using enzyme efficiency as a correction factor for total amount of enzyme used in the tests. Interestingly, using this simplified adjustment (see Figure 4 legend); the correlations between *in vitro* and *in vivo* log IC_50_ were improved for all compounds (Fig. 4B). However depending on the Ki and Km values displayed by each mutant, the relationship between IC_50_ and amount of enzyme can be complex and a determination of these values through classical enzymology would be required to further improve the correlation factor applied here [51]. In addition to enzyme efficiency, stability upon catalysis [42], [50] and modification of preferred substrate [43] are factors which might also have an influence on the establishment of resistance *in vivo*.

### *In vivo* and *in vitro* sensitivity comparison between ectopic transformants and homologous recombinants carrying resistant alleles

**Figure 5:**
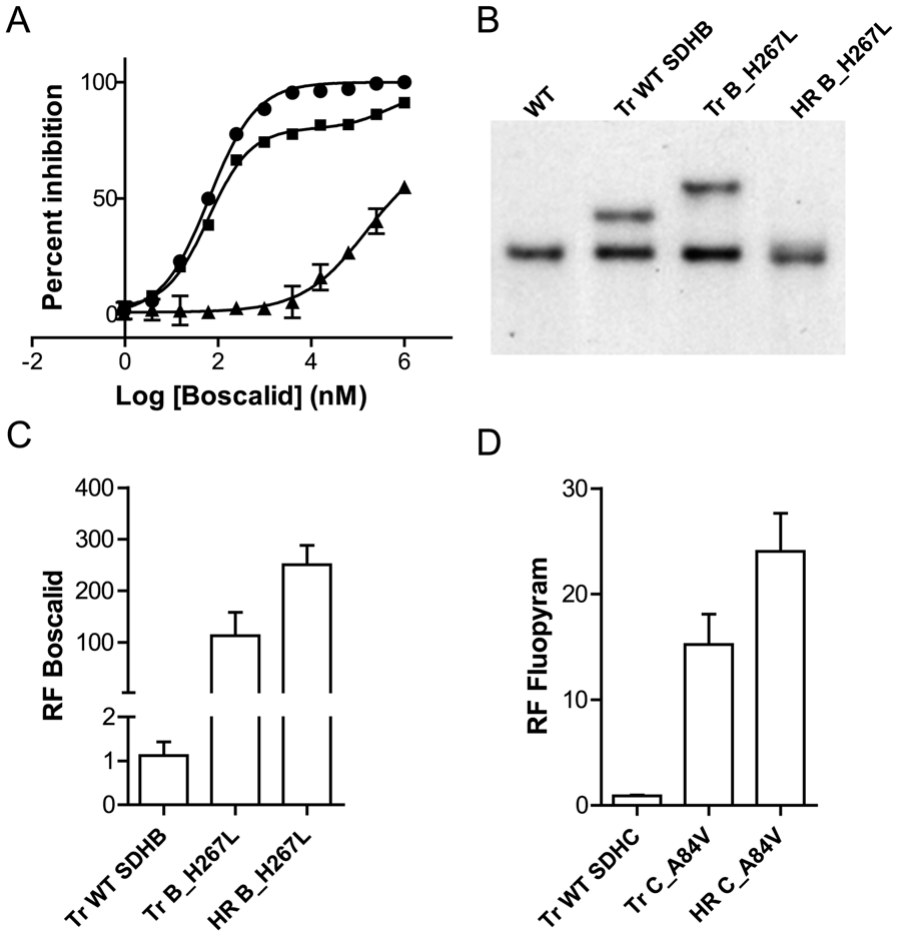
Comparison of the resistance phenotypes displayed by transformants of the SDHB or SDHC subunits. Two types of transformants were created, (i) Tr strains where the genes encoding either SDHB or SDHC subunits were ectopically inserted under the control of a GPDA promoter, (ii) HR strains where the WT gene was replaced by a mutated version in its original genomic context. A: SDH inhibition displayed by mitochondrial extracts as measured with the succinate: Q_0_/DCPIP reduction test in the presence of varying concentrations of Boscalid. Fitted curve is monophasic with the ectopic transformant containing the WT SDHB expression cassette (black dots) and with the homologous recombinant strain carrying the SDHB_H267L mutation (HR B_H267L, black triangles). Inhibition is biphasic with the ectopic transformant containing the SDHB_H267L expression cassette (Tr B_H267L, black squares). B: Southern blot of genomic DNA extracted from (left to right), the WT (IPO323), one ectopic transformant carrying the WT_SDHB expression cassette, one ectopic transformant carrying the SDHB_H267L expression cassette and signal obtained with one SDHB_H267L homologous recombinant. Boscalid (C) and Fluopyram (D) resistance phenotypes displayed by ectopic and homologous recombinants transformants. In both cases when additional WT SDHB (Tr_WT_SDHB) and SDHC (Tr_WT_SDHC) were inserted ectopically, no significant increase in resistance was observed. When an additional mutated copy of SDHB (Tr_SDHB_H267L) or of SDHC (Tr_SDHC_A84V) was inserted ectopically, significant resistance to the compounds was observed, clearly indicating a dominant effect of the mutated alleles. However, in the homologous recombinant strains where only the mutated subunit SDHB (HR_SDHB_H267L) or SDHC (HR_SDHC_A84_V) was present a further increase in resistance is observed. Whiskers represent minimum and maximum RFs obtained, boxes represent 95% confidence interval and bars value of the median. The data presented correspond to average values of duplicated tests with four independent events of each kind.

To explore the influence of SDH mutations *in vivo* in more detail, we introduced ectopic expression cassettes in the *M. graminicola* IPO323 WT strain creating a strain which is artificially heterozygous for a particular *SDH* gene (Materials and Methods, Fig. 5B). Corresponding homologous recombinant (HR) strains where the wild type SDH gene was replaced with the mutant form were also generated for comparison. These HR events were fully characterized molecularly, confirming the presence of a unique mutation in the whole genome differentiating the WT strain (IPO323) from its corresponding homologous recombinants (cf Materials and Methods). The ectopic transformant Tr_SDHB_H267L expresses a mixture of both sensitive (SDHB_WT) and resistant (SDHB_H267L) SDHB containing enzymes. As a result, the mitochondrial Boscalid inhibition curve is biphasic reflecting the two populations of enzymes present (Fig. 5A). The WT enzyme is inhibited at low doses but mitochondrial activity is maintained at higher doses indicating the contribution of mutated enzyme to the mitochondrial activity in this transformant. Interestingly, in the ectopic transformant expressing SDHB_H267L (Tr B_H267L), the total SDH activity which can be attributed to the mutated enzyme (see Fig. 5A, remaining activity at WT inhibiting concentrations) is much lower than the one attributable to the WT SDH. Despite its lower contribution to the total SDH activity in the mitochondria, clear dominance of the mutated allele can be seen *in vivo* using liquid culture tests (Fig. 5C). However, resistance levels reached by TrH267L strains remained significantly lower (unpaired *t* test, p<0.01) compared to the one reached by the corresponding homologous recombinant HR_H267L strains. This effect was not restricted to SDHB, as similar results were obtained with the SDHC_A84V mutation towards Fluopyram (Fig. 5D, also p<0.01). These results may indicate competition between the WT and mutated subunit for assembly into the functional enzyme in the mitochondria of the ectopic transformants. This assumption is further supported by the observed carboxamides oversensitivity displayed by ectopic transformants expressing non functional subunits (data not shown, [48]). This would explain the weaker phenotype seen with the ectopics carrying less mutated enzyme compared to the pure mutant.

Taken together, our results confirmed that total SDH activity level remaining upon carboxamide inhibition is the driver for cell survival. Despite the major loss in SDH enzyme efficiency observed in many of the mutants compared to the WT (Table 4), here we show that very low levels of activity are sufficient to confer survival upon carboxamide treatment (compare Fig. 5A and Fig. 5C). This suggests that *in vivo*, carboxamide inhibitors have to almost completely block SDH enzymatic activity in order to deliver their fungicidal effect.

### Generation of homologous recombinants for most relevant mutation types

**Figure 6:**
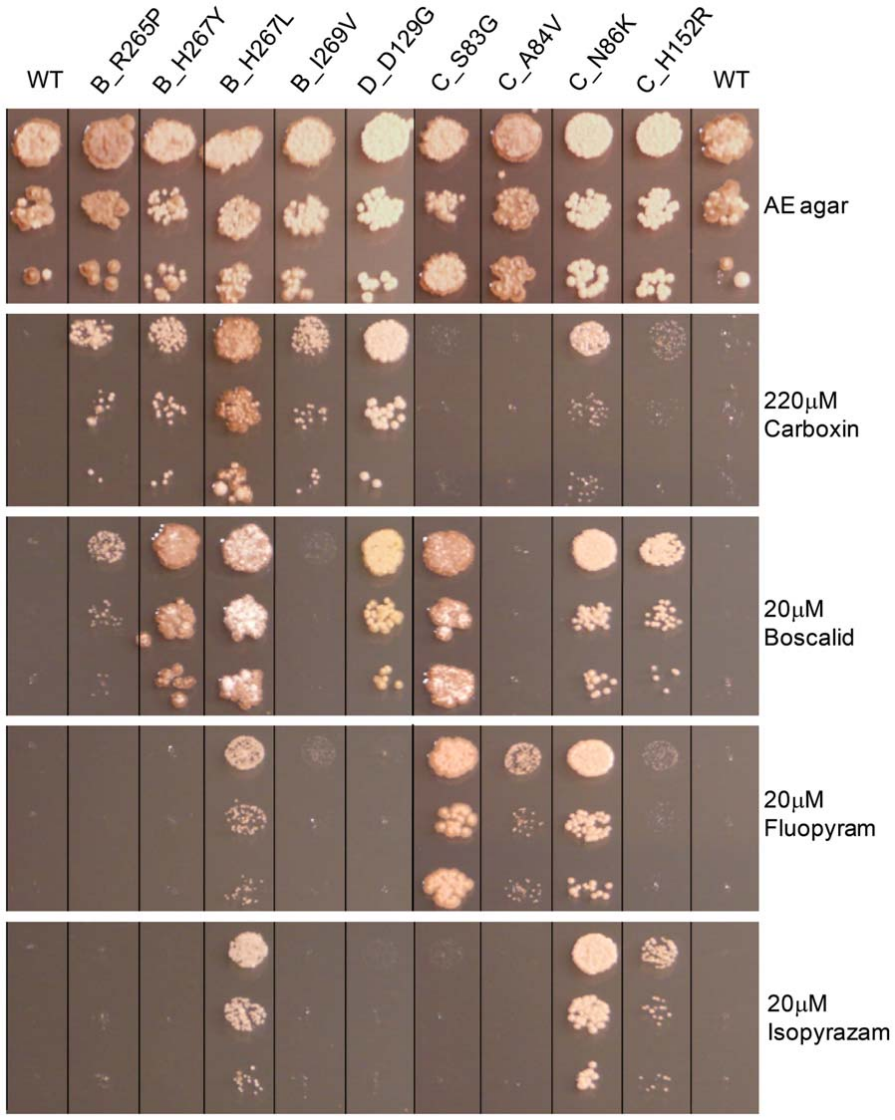
Comparative growth of homologous recombinant strains on various carboxamide amended media. All strains were grown on AE agar supplemented (or not) with the indicated active ingredient (final concentration indicated). Cell suspensions obtained from 4 days old V8 agar plates were serially diluted by 10 fold factor steps in liquid AE and 2 ul were spotted onto the plates. Pictures were taken 10 days post inoculation for carboxamides amended plates and 6 days post inoculation for AE plate. Pictures were taken from same plates and reordered for visualization.

To fully explore a potential fitness cost conferred by SDH mutations we generated homologous recombinant strains for some of the strongest and most frequent substitutions types. The following substitutions were selected for further analysis: SDHB_R265P and SDHB_I269V uniquely obtained upon Carboxin selection at MSC, SDHB_H267Y most frequent substitution occurring mutant following selection at the MSC for Carboxin and Boscalid, SDHB_H267L leading to high resistance levels towards all classes *in vitro*, SDHC_S83G leading to the strongest Fluopyram and Boscalid resistance *in vivo*, SDHC_A84V being the most frequent mutation occurring upon Fluopyram selection at MSC and conferring specific resistance to this compound and SDHC_N86K and SDHC_H152R, strongest and most frequent mutations found with Isopyrazam selection. As shown in figure 6, the 9 HR strains displayed similar growth on non selective AE media even though some pigmentation difference can be noticed. Similar growth behaviour was maintained on a minimal media (data not shown). The strength of growth obtained on SDHIs amended media correlated well with the *in vivo* resistance factors obtained using their corresponding UV mutants in liquid culture test (Table 3) validating these target mutations fully conferred observed cross resistance profiles.

### SDH mutants are not hypersensitive towards oxidative stresses

**Figure 7:**
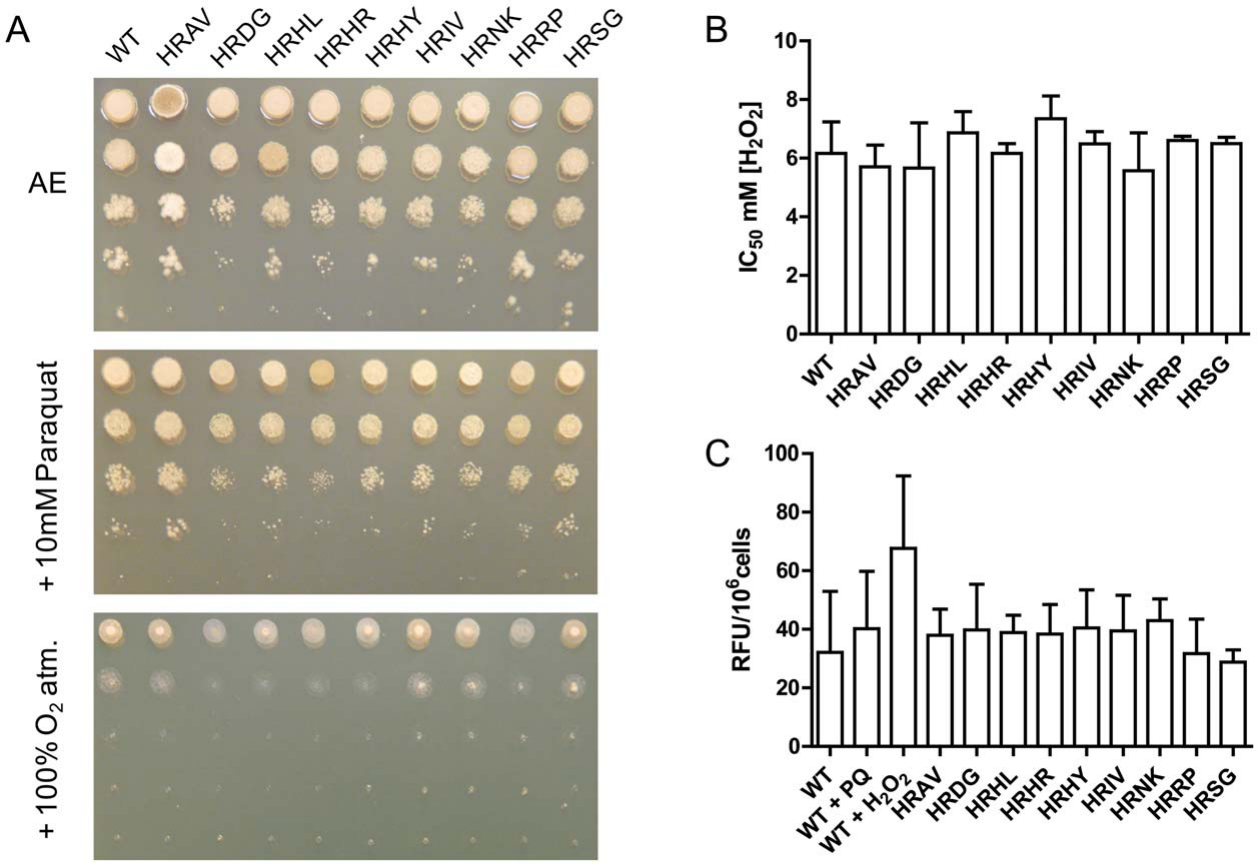
Absence of a major oxidative stress related fitness penalty in *M. graminicola* homologous recombinant strains carrying Qp site mutations. A: Respiratory growth of wild type and mutants in the presence of oxidative stresses. Upper panel, AE agar plate growth, middle panel: AE agar supplemented with 10 mM Paraquat, lower panel: AE agar plates were placed in a constant 100% atmosphere. All plates were incubated in same room at room temperature for 6 days. B: Sensitivity towards hydrogen peroxide in liquid AE media. Values presented are the averages from replicates within 3 individual experiments. C: Evaluation of mitochondrial ROS produced *in vivo* as determined with MitoSOX™ Red fluorescent indicator. Evaluations based on values from over 3 replicates within 3 individual experiments. +PQ corresponds to 1 mM Paraquat supplementation during the incubation period, +H_2_O_2_ correspond to 10 mM H_2_O_2_ supplementation during the incubation period. WT (IPO323), and homologous recombinant strains HRAV: SDHC_A84V, HRDG: SDHD_D129G, HRHL: SDHB_H267L, HRHR: SDHC_H152R, HRHY: SDHB_H267Y, HRIV: SDHB_I269V, HRNK: SDHC_N86K, HRRP: SDHB_R265P, HRSG: SDHC_S83G.

During catalysis, the Qp site of SDH is responsible for the 2 step electron transfer from the [3Fe-4S] iron sulphur cluster of SDHB to the ubiquinone substrate. The reaction involves the formation of a transient ubisemiquinone radical which is stabilized by electron transfer to the heme [13]. SDH Qp site mutants of *Saccharomyces cerevisae* and *E. coli* have been reported to display increased superoxide radical formation [38], [41], [52]. SDH Qp site mutations can also affect lifespan of organisms by causing a continuous oxidative stress which has been associated to premature aging and tumor formation [52], [53], [54], [55]. Mutations of the Qp site residues corresponding to SDHB_P220, SDHC_A84 and SDHD_D129 in *M. graminicola* have been shown to confer increased ROS production and oxidative stress hypersensitivity in other organisms including *E. coli* [38], *Caenorhabditis elegans* [52], [53], and *S. cerevisae* [41]. To assess whether carboxamide-selected Qp site mutations isolated in this study display a similar impact on oxidative stress sensitivity in *M. graminicola* we compared sensitivity of a subset of the generated homologous recombinant strains to various oxidative stresses. Two superoxide inducing conditions were used, treatment with 10 mM Paraquat (methyl viologen), a herbicide reported to induce oxidative stress through the generation of superoxide anions and a continuous 100% oxygen atmosphere simulating hyperoxia. Paraquat supplemented media had a similar impact on growth of both the homologous recombinant strains and the WT (Fig. 7A, middle panel). UV strains representative of all mutations types (excluding SDHB_P221 mutants, strongly impaired in growth) were tested using same conditions but did not display any clear difference in growth (data not shown). Similarly, hyperoxia simulated by a continuous 100% oxygen atmosphere showed a very minor impairment in growth for the SDHB_R265P and SDHD_D129G mutants (Fig. 7A, lower panel). This suggests that all carboxamide selected SDH mutations have no or a minor influence on ROS production in *M. graminicola in vivo*. When hydrogen peroxide was used as a further oxidative stress inducer and the data subjected to analysis of variance, again no significant shift in the sensitivity towards this oxidizing agent could be detected across WT and HR strains (p>0.1) (Fig. 7B). This result is further supporting these carboxamide-selected *M. graminicola* SDH mutations have a minor or no oxidative stress related fitness penalty *in vivo*. To evaluate ROS production in the mitochondria of SDH Qp mutants *in vivo*, we used the intracellular ROS indicator MitoSOX™ Red (Molecular Probes). As could be expected from the lack of hypersensitivity to oxidative stresses in previous *in vivo* tests, comparison of our subset of homologous recombinant strains showed no clear evidence for a difference across the WT and the target mutants (p>0.1) (Fig. 7C). However, in all conditions tested, fluorescence intensity remained very low, even hydrogen peroxide and Paraquat driven changes in fluorescence signal were not significantly higher than WT. Poor signals were also obtained with the cytosolic ROS marker dihydroxyethidium bromide (DHE, Molecular probes) (data not shown). These results may be caused by a poor uptake of these small molecules by the fungal cells or highlight a very good defence against oxidative agents in this pathogen.

### Impact of SDH mutations on necrosis symptom development *in planta*

**Figure 8:**
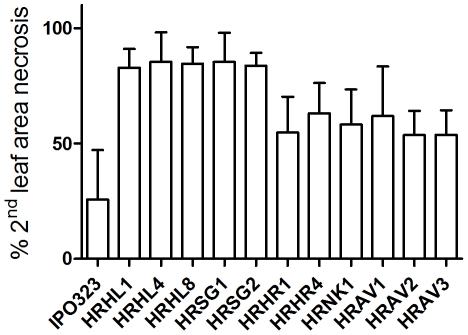
Comparison of *in planta* necrosis symptoms displayed by individual homologous recombinant strains carrying mutations conferring resistance to carboxamides. Data presented correspond to the individual means (average of 12 leaves each) of 3 individual experiments ± S.D. Numbering corresponds to individual homologous recombinants strains carrying: HRHL1, 4 and 8: SDHB_H267L substitution, HRSG1 and 2: SDHC_S83G substitution, HRHR1 and 4: SDHC_H152R substitution, HRNK1: SDHC_N86K substitution, HRAV1, 2 and 3: SDHC_A84V substitution.

In order to evaluate the impact of SDH mutations on *in planta* growth, a smaller subset of the recombinant strains was compared for necrosis symptoms displayed *in planta*. For comparing the diverse SDH mutants we used various homologous recombinant strains carrying SDHB_H267L, SDHC_H152R, SDHC_N86K and SDHC_A84V mutations. Homologous recombinant strains were similarly inoculated on the sensitive WT plant Riband and *in planta* symptoms assessed visually 14 days after inoculation (Fig. 8). The data were subjected to analysis of variance which detected clear evidence of differences amongst strains (p<0.01). The statistical significance of differences between the WT and each of the HR strains was determined using an LSD test which showed that the HR strains displayed significantly higher symptomology compared to the WT (p<0.05). Furthermore, no difference was seen between strains carrying a similar SDH mutation (p>0.1 in all cases) confirming these *in planta* phenotypes are fully SDH driven.

Interestingly, comparison of these results with those in table 4 shows that the magnitude of necrosis symptoms are not correlating to SDH activity impairment as quantified by the succinate: Qo/DCPIP activity test. For example the SDHC_S83G mutants displayed a similar increase in symptomology compared to the SDHB_H267L mutants in spite of their different impact on enzyme efficiency (mutations conferring 59% and 13% residual activity respectively). Similarly the SDHC_H152R mutants (22% residual activity) displayed similar necrosis size compared to SDHC_A84V mutants (84% residual activity), and to the SDHC_N86K mutant (41% residual activity). This result suggests mutations in the SDH enzyme have an impact on the biology of the pathogen.

## Discussion

In this study, we developed a better understanding of the binding properties and resistance mechanisms for a range of new carboxamides recently introduced as crop protection fungicides. The different biological spectrum displayed by the new carboxamides demonstrates that an incredibly broad range of biological specificities can be developed from a single core structure. By comparing enzyme inhibition and biological profiles, we have previously found that biological activity is primarily driven by the affinity of a molecule to the SDH enzyme in targeted organisms [48]. Poor conservation in residues belonging to subunits SDHC or SDHD surrounding the Qp site of SDH is observed across fungal species. One of the challenges in delivering good agrochemical solutions from carboxamide chemistry has been to overcome this variation in order to deliver an effective balance between binding efficacy and fungal spectrum. Partly because of this wide structural variation in the target enzyme, a unique solution enabling the control of all fungal pathogens could not be found. Therefore, further SDHIs that display additional fungicide spectrum might be introduced in the coming years.

Our mutagenesis study led us to identify 27 different substitution types affecting 18 positions in 3 of the 4 subunits encoding the Qp site of the target SDH enzyme. The pattern and frequency of mutations selected was found to be highly dependent on the compound used for selection. Accordingly, sensitivity profiles are substitution dependent, as a result of specific interaction of different classes of inhibitors to specific structural features of the enzyme. The large majority of the mutations lead to a sensitivity decrease across all carboxamides *in vivo*, but the level of reduced sensitivity shows a high degree of variation across the carboxamide/substitution pairs studied. More practically, this suggests that the use of carboxamides of different structures to control the same pathogens will strongly influence the nature and composition of the mutant population in the field as was found in *A. alternata* field trials [56].The nature of carboxamide-selected *M. graminicola* target mutations found in the laboratory display striking similarities with the mutations found in *B. cinerea* field populations following several years of Boscalid usage [30], [32]. Among the various substitutions detected in *B. cinerea* field isolates the SDHB_H272Y/L, SDHB_P225L/T and SDHB_N230I substitutions correspond to SDHB_H267Y/L, SDHB_P220L/T and SDHB_N225I carboxamide-selected substitutions respectively in *M. graminicola*. This shows that carboxamide resistance can be conferred by similar substitutions at similar positions within the structure of the SDH enzyme across species. Contrasting with the SDHB_P225L/T substitutions conferring high levels of resistance towards Boscalid and Fluopyram in *B. cinerea* ([30], Syngenta, unpublished result). In our screen, the *M. graminicola* SDHB_P220L/T substitutions were obtained upon Fluopyram selection only and conferred limited resistance towards this active ingredient as well as weak Boscalid resistance (Table 3). Therefore, subtle differences in the structure of the Qp site of SDH within targeted organisms are likely to influence the nature of substitutions conferring resistance to a given carboxamide and this highlights the problems in extrapolating resistance prediction from one pathogen to another. This is further exemplified by the different substitution patterns and associated resistance factors displayed by *A. alternata* pistachio field isolates after a few years of Boscalid usage [33], [34]. Despite the difficulties in extrapolating between species some key conserved interactions are beginning to emerge. For example, Fluopyram hypersensitivity is observed in SDHB histidine to tyrosine Qp site mutants in a range of species including *M. graminicola* (SDHB_H267Y), *B. cinerea* (SDHB_H272Y) and *A. alternata* (SDHB_H277Y) ([30], [56], Syngenta, unpublished results). A similar substitution may also explain the similar negative cross resistance behavior observed in some Boscalid resistant isolates of *C. cassiicola* and *P. xanthii* [36]. Using the homology model developed in this study, a possible explanation for this conserved negative cross resistance was proposed. In the WT enzyme, a key H-bond interaction may occur between the rotated histidine of the Qp site and the acceptor group of Boscalid. This crucial interaction for binding is removed by the tyrosine substitution which consequently impairs Boscalid binding in the mutant. Contrastingly, Fluopyram which has no H-bond acceptor group does not rely on this specific interaction for binding and is then unaffected by the histidine to tyrosine substitution. Further confirming this assumption, compound A which also lacks a H-bond acceptor group gives also better control of the *M. graminicola* SDHB_H267Y mutant compared to the WT (data not shown). Given the degree of conservation for cross resistance profiles seen with this particular mutant it seems that the depicted interaction is consistently conserved across species.

Using transformation we evidenced that the remaining SDH activity present in the cells at a given inhibitor concentration is responsible for survival. Interestingly, very low levels of SDH activity were sufficient for the establishment of resistance, as confirmed by the selection of substitutions leading to over 90% loss in activity. This suggests that for each mutant, *in vivo* survival upon carboxamide treatment is a balance between a negative impact brought by decreased enzyme activity/stability caused by substitutions affecting the Qp site and a positive one brought by poorer binding of carboxamide inhibitors resulting in weaker inhibition of the enzyme. From a cellular perspective and considering the central role of SDH for energy production, it seems logical that the remaining SDH activity, which is necessary to maintain an active TCA cycle, is the driver for survival.

A balance between substrate and inhibitor binding would explain why some highly conserved residues of the Qp site which are predicted to be essential for carboxamide inhibitor binding in the tridimensional model were neither found substituted in our screen nor reported yet in field populations. Notably the fully conserved Qp site residues SDHB_W224 and SDHD_Y130 which are predicted to hydrogen-bond to the amide oxygen of carboxamides [15], [40]. In agreement with the essential involvement of the conserved SDHD tyrosine in the establishment of a crucial hydrogen bond to one quinone oxygen, *E. coli*, SDHD_Y83F and *S. cerevisae* SDHD_Y89F substitutions impair 85% and 95% of the ubiquinone reductase activity respectively [13], [57]. We introduced the SDHD_Y130F substitution in the *M. graminicola MgSDHD* gene using site directed mutagenesis and found that ectopic transformants expressing SDHD_Y130F are more sensitive to carboxamides compared to the WT (data not shown). The absence of any mutation at this residue for all carboxamides tested might indicate that substitutions at this position could not confer selective advantage in the balance between catalysis and inhibition.

Because SDH enzyme activity was impaired in all mutants we expected to find some degree of fitness penalty *in vivo*. Furthermore, similar Qp site substitutions have been shown to have biological influence on the lifespan of organisms through the increased production of ROS by the mutated SDH enzyme [38], [41], [52], [53], [54], [55]. To primarily address this and to prevent the likely interference caused by mutations in other genes in UV mutants, we generated homologous recombinants to some of the most relevant substitution types. The absence of any significant growth defect in our carboxamide-selected Qp site mutants and homologous recombinant strains suggested that the likely increased ROS production by the mutated enzyme was not exceeding the capacity of the antioxidant defense system in *M. graminicola*. One explanation for this result may be that our initial selection for carboxamide resistance is strongly biased against the selection of mutants which display high level of oxidative stress. This could occur if the presence of ROS producing mutations impacted growth as shown with many yeast Qp site mutants on non-fermentable media [38], [41]. Impaired growth could have resulted in the absence of identifiable colonies at the point of colony selection. Carboxamide-selected Qp site substitutions were all leading to decreased ubiquinone reductase activity suggesting that, at least in some cases, the stability of the transient ubiquinone semiradical generated during the course of the reaction might be affected and thus lead to locally increased ROS production [38]. Therefore, the absence of strong oxygen hypersensitivity phenotypes is surprising and may be explained by a combination of factors. First the specific activity displayed by *M. graminicola* SDH is 10 times lower compared to that of *S. cerevisiae* SDH [41]. More generally, *M. graminicola* is a slow growing pathogen and might have a much lower mitochondrial ROS production rate compared to fast growing fungi like *S. cerevisiae*. Secondly, *M. graminicola* is a hemibiotrophic pathogen and therefore has to survive plant induced oxidative burst in colonized plant tissue. The pathogen requires and induces an exceptionally efficient ROS detoxification enzyme toolset in order to survive the necrotrophic stage of its lifecycle [58]. In fact it has been suggested that ROS production by *M. graminicola* itself and ROS signalling may also contribute to pathogenicity [58], [59]. Therefore, the combination of a slower turnover with the presence of an extraordinary large enzymatic toolset capable of defend against ROS in this species might lead to the very poor impact of oxidative stresses towards carboxamide-selected Qp site mutants when compared to other species. These hypotheses are further supported by the poor fluorescence signals obtained with intracellular ROS indicators showing that no significant ROS accumulation occurs in both WT and mutants. *In vitro* tests with a purified mitochondrial fraction will be required to measure the potentially increased production of ROS by the mutated *M. graminicola* SDH enzyme in more detail [38], [41], [52]. This and further work would be required to understand the marked differences in ROS generation and sensitivity between *M. graminicola* and other species.

In our test conditions, *in planta* virulence tests performed with some of the HR strains showed that some of the SDH mutations can lead to increased symptoms in the absence of carboxamide treatment. The reason for this remains unclear, but the increased symptomology observed in the climatic room is unlikely to result in increased fitness in the field as nature would have selected such SDH variants during the course of evolution if it was the case. One explanation for the observed phenotype is that the presence of less efficient SDH enzyme might lead to increased intracellular levels of succinate as observed in yeast mutants [41] and in *M. graminicola* WT upon treatment with sub-lethal doses of carboxamides (data not shown). In yeast, perturbations of the succinate dehydrogenase function have a large impact on metabolism. Moreover, it was shown that succinate dehydrogenase mutations displaying similar impairment in quinone reductase activity can lead to very distinct metabotypes [60]. The metabolic impact of impairments of the *M. graminicola* SDH enzyme may lead to developmental effects on *in planta* growth and symptoms development as complex regulation of metabolic fluxes have been shown to play a major role in the infection process [58], [61], [62]. Necrotrophic growth involves massive expression of transporters and of enzymes involved in primary metabolite uptake and degradation and it is very likely to involve secondary metabolites (small molecules toxins) production [58], [61], [62]. Modifications in primary metabolism might have an influence on this metabolic switch and potentially increase secretion of small toxin molecules thus explaining the observed increased symptomology. Even though the reasons for this increased symptomology *in planta* remain elusive, our data indicate that SDH mutations can have an influence on the biology of the pathogen. Aside additional fitness penalty and resistance studies to be performed *in planta*, the question as to know whether this phenomenon is influenced by ROS or TCA metabolites or both will require further evaluations now rendered possible by the homologous recombinants generated during the course of this study.

Finally, our data clearly shows that a combination of factors might have to be considered for a diagnosis of mutations likely to occur in the field. In our mutagenesis screen, high resistance factors, frequency of occurrence and maintained *in planta* growth point towards some mutations for which sensitive molecular tests will be designed and applied in the monitoring of field populations. These results combined with the resistance situation in other pathogens further stress the importance of a proper anti-resistance strategy for the SDHIs fungicides. At this point in time and in order to prolong the efficacy of this class of fungicide in wheat, recommendations include restrictions in the number and timing of applications as well as the mandatory usage of mixtures (see www.FRAC.info).

## Materials and Methods

### Strains, media and culture conditions

*M. graminicola* isolate IPO323 was used for all experiments [63]. The isolate was inoculated from stocks stored in liquid nitrogen onto solid V8 agar [64]. Yeast like cells were harvested from these plates and used immediately as an inoculum for all experiments. The following media were used throughout: Aspergillus minimal medium (AMM) [64]; TSM40 (4 g glucose, 10 g malt extract, 4 g yeast extract, pH 7.0); AE medium [23]; induction medium (IM) [64]; potato dextrose agar (PDA) [64]. DH5α or ccdB survival cells (Invitrogen) were used for the maintenance of plasmids in *E. coli*. *Agrobacterium. tumefaciens* strain EHA105 [65], [66], was used for maintenance of constructs and for *A. tumefaciens* mediated transformation.

### Carboxamides, mutagenesis and UV mutant isolation

Minimal selective concentration (MSC) is the minimal concentration of carboxamide necessary to fully inhibit growth of the wild type IPO323 *M. graminicola* strain after inoculation of AE agar plates with 10^7^ cells followed by incubation for 2 weeks at 20°C in the dark. Selection plates were produced after adding 1% of a 100× DMSO solution of the active ingredients to pre-melted AE-agar. A higher concentration corresponding to 5× MSC was applied with Carboxin, Isopyrazam, Fluopyram and Boscalid and 2.5× MSC with the Isopyrazam pyrrole analogue compound A.

Cells were harvested from 4 days old V8 agar plates and conidial cells were suspended in liquid AE. 10^7^ cells were spread onto carboxamide amended AE agar plates and mutagenized using UV Stratalinker 2400 (Stratagene) with 75 mJ cm^−2^ or 50 mJ cm^−2^. These UV treatments produce lethality of 80% and 50% respectively. Selection plates were incubated in the dark at 20°C for 14 days. Visible colonies were picked from the primary selection plate and re-grown on fresh selection media for 2 additional weeks under the same conditions.

### Molecular characterization of UV mutants

Strains were grown in 5 ml liquid TSM40 on a reciprocal shaker (220 rpm, 20°C) for 5 days prior to genomic DNA extraction using a Qiagen mini plant kit (Qiagen). PCR primer design was based on the *M. graminicola* IPO323 genome sequence [63]. The four SDH-encoding genes were PCR amplified and sequenced using primers listed in Table S1. For comparison purposes, SDHB, SDHC and SDHD proteins sequences were retrieved for a number of different species as listed in supplemental data section (Table S2).

### Production of *M. graminicola* transformants

For ectopic integration of SDH subunit expression cassettes, the multisite binary pNOV2114_gateway vector was used with the *A. nidulans* gpda (glyceraldehyde-3-phosphate dehydrogenase) promoter to drive the expression [64]. Wild type or mutated *MgSDH* gene were amplified from genomic DNA using PfuTurbo-DNA polymerase (Stratagene) and two rounds of amplification with primers listed in table S1. The attB PCR fragments were cloned into pDONRzeo plasmid (Invitrogen). Finally, multiple gateway reaction was performed using multisite Gateway cloning (Invitrogen) to produce pNOV_gpda_*MgSDHB*_TrpCter, pNOV_gpda_*MgSDHB-H267L*_TrpCter, pNOV_gpda_*MgSDHC*_TrpCter, and pNOV_gpda_*MgSDHC-A84V*_TrpCter plasmids.

For homologous recombination, genomic fragments containing part of the *SDH* gene without the start codon but containing the mutation(s) leading to the specified amino acid substitution plus ∼800 bp down-stream of the gene were PCR amplified using primers described in Table S1 and cloned into the multiple cloning site of pNOV2114 vector [67], [68]. Oligonucleotides were designed to avoid in-frame start codons upstream of the cloned fragment.

*M. graminicola* transformation procedure has been described previously [64]. For isolation of homologous recombinants, selection of transformants was performed on AE agar supplemented with an appropriate carboxamide. Three rounds of selection were applied. Absence of the start codon in the homologous recombination constructs ensured low frequency of functional expression from ectopic insertions.

### Molecular characterization of transformants

For ectopic transformants, primer pairs were designed to screen for the presence and completeness of the gpda promoter, *MgSDH* gene and hygromycin resistance gene. For homologous recombinants, primer pairs were generated to verify the unique presence of the mutated copy using differential restriction profiles displayed by the mutated allele versus the non mutated allele. In addition primer pairs were designed to screen for the absence of T-DNA downstream of the integrated fragment. All strains which passed PCR verification were sequence verified. All PCR primers and details mentioned above are listed in Table S1.

The copy number of the SDH gene in the transformed strains was assessed by Southern blot. Hybridization probes were produced by PCR amplification of the SDH gene of interest and labeled using the AlkPhos Direct™ system (GE Healthcare). Genomic DNA (∼10 µg) were digested using BamH1 for *SDHB* and *SDHD* transformants; BamH1/EcoR1 for *SDHC* transformants. Digested DNA was electrophoresed and subsequently blotted and UV cross-linked to Hibond™ Nylon membranes (GE-Healthcare) [69]. Hybridization was performed using AlkPhos Direct™ reagents (GE-Healthcare). A hybridization temperature of 55°C was used for labeling all three *SDH* genes, followed by washes at 55°C (*SDHC* and *SDHD*) or 60°C (*SDHB*). Luminescence signal was generated with CDP-Star (GE-Healthcare) and visualized on Kodak Biomax light films (Sigma).

### Mitochondria isolation

Strains were grown in 1 liter TSM40 units. Cultures were inoculated at 0.05 OD_600 nm_ and grown on a reciprocal shaker (110 rpm, 22°C) for 5 days. Cells were harvested by vacuum filtration and disrupted in liquid nitrogen using a mortar and pestle. Resultant powder was re-suspended to 10% w/v in mitochondrial extraction buffer (10 mM KHPO_4_ pH7.2; 10 mM KCl, 10 mM MgCl_2_, 0.5 M sucrose, 0.2 mM EDTA, 2 mM PMSF). Extract was clarified by centrifugation (5,000 g, 4°C for 10 minutes, 2 times), intact mitochondria were then pelleted at 10,000 g for 20 minutes at 4°C. The pink-reddish pellet was re-suspended in a small volume (1 ml for 10 grams initial fresh weight) of extraction buffer. This volume was transferred to conical 1.5 ml tubes and re-centrifuged, 10,000 g for 10 minutes at 4°C. The pellet separates into two layers; the upper reddish colored being easily re-suspended by pipetting is composed of nearly pure mitochondria as assessed by microscopy. Subsequent sucrose gradient centrifugation did not improve specific activity of the mitochondrial fractions; therefore the purification procedure was stopped at this stage. Mitochondrial suspensions were brought to a final concentration of 10 mg ml^−1^ in extraction buffer and stored at −80°C until used. SDH activity was found to remain stable for months.

### Enzyme assays

Mitochondrial suspensions were incubated at 30°C for 30 minutes in extraction buffer supplemented with 10 mM succinate prior to enzymatic measurements.

Succinate: ubiquinone/DCPIP measurements were performed by adding 5 µl of pre-activated mitochondria to 100 µl assay buffer (50 mM Phosphate-Na pH 7.2, 250 mM sucrose, 3 mM NaN_3_, 10 mM succinate) supplemented with 140 µM Dichlorophenolindophenol (DCIP) and 1 mM of 2,3-dimethoxy-5-methyl-1,4-benzoquinone (Q_0_), from a 0.1 M DMSO stock solution. A reaction plate was equilibrated for 10 minutes at 30°C before starting the reaction. DCPIP reduction was conducted at 30°C and monitored at 595 nm using a HTS7000 microtiterplate reader (Perkin Elmer). For calculations a molecular extinction coefficient of ε = 17 mM^−1^ cm^−1^ was used [70].

The succinate: PMS (Phenazine methosulfate) coupled reduction of MTT ((4,5-dimethylthiazol-2-yl)-2,5-diphenyltetrazolium bromide) measurements were performed using final concentrations of 400 µM PMS and 150 µM MTT freshly diluted in assay buffer (50 mM Phosphate-Na pH 7.2, 250 mM sucrose, 3 mM NaN_3_, 10 mM succinate). Reactions were started with the addition of 5 µl of preactivated mitochondrial suspension to 100 µl of the reaction mixture at 30°C using an experimental setup similar to DCPIP measurements. The MTT reduction was monitored by reading the absorbance increase at 570 nm using a HTS7000 microtiterplate reader (Perkin Elmer). For calculations a molecular extinction coefficient of ε = 21 mM^−1^.cm^−1^ was used [71].

In the succinate: ubiquinone/DCPIP test, activity displayed by some mutants was so low that an adjustment for enzyme activity was necessary for accurate IC50 determination. For this reason, all mitochondrial suspensions were adjusted to a similar initial velocity (1 OD_595 nm_ hour^−1^) following conditions described above. Inhibitor concentrations ranged between 1 mM and 0.95 nM with uniform 4× dilution factor steps (11 inhibitor concentrations+DMSO control). 96 wells plates were pre equilibrated at reaction temperature (30°C) for 10 minutes before the reactions were started by the addition of 5 µl preactivated *M. graminicola* mitochondrial suspension. Calculated absorbance slopes (OD/hour) were used for IC50 calculations using Graphpad Prism 5.0 software non linear curve fitting against log inhibitor concentrations.

### Flavin Quantification

Fluorometric quantification of the covalent FAD of SDH was carried out as described in [72] using 1 mg of protein as starting material.

#### Comparative homology modeling

The four *M. graminicola* SQR subunits were modelled by using SQR crystal structures from three different species as templates: *E. coli* SQR co-crystallized with the natural substrate ubiquinone-2 at a 2.60 Å resolution (PDB code 1NEK) [14]; *G. gallus* (chicken) SQR in complex with the inhibitor carboxin resolved at 2.10 Å (PDB code 2FBW) [15]; *S. scrofa* (pig) SQR cocrystallized with ubiquinone-2 at 2.40 Å (PDB code 1ZOY) [16].

ClustalW [73] was used to align the individual *M. graminicola* SDH protein sequences with the bacterial (1NEK), the avian (2FBW) and the porcine (1ZOY) protein subunits. The alignments were analyzed and edited in the Prime 1.5 workflow within the Maestro environment (Schrödinger, LLC, Portland, OR) [74]. In contrast to SDHA and SDHB, the alignments of the SDHC and SDHD anchoring subunits of *M. graminicola* displayed limited identity with the sequences of the three crystallographic templates. Therefore, to further improve the alignments, the multiple template strategy together with a secondary structure prediction (SSPro4.0 [75]) was applied. In the next step, the actual three-dimensional models of each four subunits were built according to the standard superimposition and side-chain orientation of Prime protocols. The individual sub-models were then assembled by superimposition on the 2FBW structure keeping carboxin inside the Qp site as well as the heme, the FAD and malate-like intermediate inside the so-called “substrate binding site” to help in the definition of pockets and cavities. The refinement module of Prime (PLOP, Protein Local Optimization Program [76]) was used to optimize the sidechain conformations with particular attention given to the SDHB, SDHC and SDHD interfaces and other regions in the vicinity of Qp-site. Moreover default loop refinement method was performed on sections coating the Qp-site, 256–271, 226–234 on SdhB and 62–78 on SdhC subunits.

The final *M. graminicola* SQR model was optimized within the OPLS2005 force field in Prime [74], [77].

#### Pharmacophore elucidation

All SDH crystal structures were extracted from the Protein Data Bank (PDB) and superimposed onto each other. The Qp binding sites of the superimposed structures with a resolution higher than 3 Å were visually analyzed in more detail for common pharmacophoric features (PDB codes 3AE4, 3AEF, 2WQY, 2H88, 2H89, 1YQ3, 1YQ4, 2FBW, 1ZOY, 1NEK, 1NEN, 2WDQ, 2WP9, 2WU5).

#### Molecular Docking

Carboxin was manually placed in the Qp binding site of *Mg*SDH with Moloc [78] in an analogous way as observed in the carboxin *G.gallus* SQR complex crystal structure 2WQY. In a second step the protein ligand complex was minimized using Moloc MAB force field [78], allowing full flexibility for the ligand and keeping the *M. graminicola* SQR protein rigid.

Boscalid, Fluopyram, and Isopyrazam were manually docked into the *M. graminicola* SQR Qp binding site. Interactions to key residues which were determined through the pharmacophore elucidation (see results) were formed. In a second step the protein ligand complexes were minimized using Moloc MAB force field [78], allowing full flexibility for the ligands and keeping the *M. graminicola* SQR protein rigid.

#### Mutated Qp-site modeling

Mutations were generated within the *M. graminicola* SQR homology model with the “mutate residue” procedure included in Maestro (Schrödinger, LLC, Portland, OR), followed by a contact analysis involving multiple rotamers and a geometry sanitation within the OPLS2005 force field.

#### *In vivo* tests for strain sensitivity determination

Conidial cells were collected from 4 day old V8 agar plates (cultured at 18°C in the dark) using liquid AE and a cell spreader. Cell density was determined and cells re-suspended to a concentration of 5×10^5^ cells ml^−1^ in AE media. Sterile 96 wells microtiter plates with lids (Costar) were inoculated with 198 µl of this cell suspension (10^5^ cells per well) and 2 µl of inhibitor (100× in DMSO). Final inhibitor concentrations were between 0.95 nM and 1 mM with uniform 4× dilution factor steps (11 inhibitor concentrations+1 DMSO control). Plates were incubated for 6 days at 19°C in the dark before cells were re-suspended and measured for growth (OD_600 nm_). IC_50_ values were determined by non linear curve fitting on the basis of measured OD values versus log inhibitor concentrations (GraphPad Prism 5.0 software).

### *In vivo* mitochondrial ROS production assay

Conidial cells were collected from 4 day old V8 agar plates (cultured at 18°C in the dark) using 2 ml of assay buffer (PIPES 10 mM pH 7.2, 2% w/v glycerol) and a cell spreader. Cell density was determined and cells re-suspended to a concentration of 3×10^7^ cells ml^−1^ in assay prior to their incubation for 2.5 hours in the dark in the presence of 5 µM MitoSOX red (Invitrogen). After the incubation period, 200 µl aliquots of each cell suspension were distributed (3 replicates per strain) into 96well plates (AcroWell™ 96 filter plates) equipped with 0.45 µm filters (Pall life sciences). First step of centrifugation 500 g for 3 minutes eliminated non incorporated fluorescent dye. Cells were resuspended in 200 µl assay buffer and same centrifugation procedure was applied. Finally, cells were resuspended in 110 µl assay buffer and 100 µl were transferred to clear bottom/black framed 96 wells Optilux™ plates (Becton Dickinson) for measurement. Fluorescence was measured with an Enspire 2300 multimode reader (Perkin Elmer) using λ_exc_ of 510 nm and λ_em_ of 580 nm (well scan mode, 100 flashes/read, 25 reads/well).

### *In planta* pathogenicity test

Conidial cells were collected from 4 day old V8 agar plates (cultured at 18°C in the dark) using 2 ml of de-ionized water. Cell density was determined and cells re-suspended to a concentration of 10^6^ cells ml^−1^ in de-ionized water supplemented with 0.05% Tween 20. Each spore suspension was used for the inoculation of three pots of two weeks old wheat (var. Riband) plantlets (3 to 4 plants/pot). The plants were homogenously sprayed and covered with a Plexiglas chamber for incubation at 100% humidity for 48 hours at 24°C in the dark. After this incubation period, pots were placed in a climatic room with alternation periods of 8 hours darkness (18°C), and 16 hours light (24°C), with 60% constant humidity. Evaluation of symptoms was performed 16 days after inoculation by visually estimating the percentage of 2^nd^ leaf area covered with pathogen–related necrosis symptoms. Negative controls (uninfected plants) confirmed the absence of necrosis at the evaluation time.

## Supporting Information

Table S1 **Oligonucleotides list.** Oligonucleotides that were used for cloning, sequencing and primary screening of successful homologous recombination events.
(XLSX)

Table S2 **Protein identifiers and source databases used in** **figure 2** **sequences alignments.** Protein identifiers are hyperlinked to corresponding feature web page in the right column.
(XLSX)
